# IL-18 Blockage Reduces Neuroinflammation and Promotes Functional Recovery in a Mouse Model of Spinal Cord Injury

**DOI:** 10.3390/biom15010016

**Published:** 2024-12-26

**Authors:** Easmin Begum, Md Rashel Mahmod, Md Mahbobur Rahman, Fumiko Fukuma, Takeshi Urano, Yuki Fujita

**Affiliations:** 1 Department of Anatomy and Developmental Biology, Faculty of Medicine, Shimane University, 89-1 Enya-cho, Izumo 693-8501, Shimane, Japan; 2 Center for Vaccines and Therapeutic Antibodies for Emerging Infectious Diseases, Shimane University, 89-1 Enya-cho, Izumo 693-8501, Shimane, Japan; 3 mAbProtein Co., Ltd., 89-1 Enya-cho, Izumo 693-8501, Shimane, Japan

**Keywords:** spinal cord injury, neuron, glia, IL-18, neuroinflammation, inflammatory response, cytokines, neuroprotection, traumatic injury, gene expression

## Abstract

The prognosis of spinal cord injury (SCI) is closely linked to secondary injury processes, predominantly driven by neuroinflammation. Interleukin-18 (IL-18) plays a pivotal role in this inflammatory response. In previous work, we developed an anti-IL-18 antibody capable of neutralizing the active form of IL-18. This study evaluated the functional effects of this antibody in a mouse model of SCI. IL-18 expression was significantly upregulated in the spinal cord following injury. In a mouse model of SCI (C57BL/6J strain), mice were administered 150 μg of the anti-IL-18 antibody intraperitoneally. IL-18 inhibition via antibody treatment facilitated motor functional recovery post-injury. This intervention reduced neuronal death, reactive gliosis, microglia/macrophage activation, and neutrophil infiltration. Additionally, IL-18 inhibition lowered the expression of pro-inflammatory factors, such as IL-1β and the M1 microglia/macrophage marker Ccl17, while enhancing the expression of the M2 microglia/macrophage marker Arginase 1. Collectively, our findings demonstrate that IL-18 inhibition promotes motor recovery and facilitates the polarization of M1 microglia/macrophages to the M2 phenotype, thereby fostering a neuroprotective immune microenvironment in mice with SCI.

## 1. Introduction

Spinal cord injury (SCI) affects millions of individuals worldwide, often resulting in permanent neurological deficits and substantial socio-economic burdens. SCI is a complex and debilitating condition primarily mediated by pro-inflammatory cytokines, which drive the secondary injury cascade [1,2,3,4,5]. Following SCI, a series of cellular and molecular events occur, including the activation of the microglia and astrocytes, an imbalance between pro-inflammatory and anti-inflammatory responses, and the onset of oxidative stress. These processes collectively contribute to neuronal death and permanent neurological deficits. The inflammatory response is further exacerbated by microglial activation and the infiltration of immune cells, such as neutrophils and macrophages. While inflammation is essential for tissue repair, excessive and prolonged inflammation exacerbates tissue damage, resulting in further neuronal death and demyelination. Therefore, understanding the mechanisms underlying secondary injury is critical for developing therapeutic strategies to mitigate long-term damage following SCI. Advancing therapeutic interventions, such as modulating neuroinflammation, offers significant promise, not only for improving patient quality of life but also for reducing the healthcare costs associated with long-term care and rehabilitation [6,7].

Increased production of cytokines from the interleukin-1 (IL-1) family is well-documented, highlighting the pivotal role of this cytokine in triggering SCI-induced inflammatory processes [8,9,10]. IL-18 is a potent mediator of inflammation that initiates and amplifies various effects associated with innate immunity, tissue injury responses, and microbial invasion [11,12,13]. Elevated IL-18 expression has been reported in a variety of diseases [14]. IL-18 binding protein (IL-18BP) acts as a natural antagonist by binding to the active form of IL-18, preventing its interaction with the IL-18 receptor complex. Elevated IL-18BP levels have been observed in numerous inflammatory conditions, and its therapeutic potential has been demonstrated in preclinical models of neuroinflammation and traumatic brain injury [15,16]. These findings underscore the importance of targeting the IL-18 signaling pathway to mitigate inflammation-induced damage. Despite extensive research on the biological roles of IL-18, challenges remain to the development of effective therapeutic interventions targeting this cytokine.

We previously developed a monoclonal antibody against IL-18 that specifically recognizes a neoepitope of caspase-1/4-cleaved IL-18 without binding to the inactive precursor IL-18 [17]. Monoclonal antibodies are generally more stable and specific, reducing the risks of unexpected safety issues compared to other therapeutic approaches. Thus, this antibody represents a promising candidate for treating human diseases involving IL-18. Elevated IL-18 levels in the blood have been reported in various conditions, including COVID-19, pulmonary diseases, and type II diabetes [18,19,20,21]. Furthermore, we evaluated the functional effects of this antibody in models of human inflammatory diseases, such as colitis [22,23]. However, the mechanisms and effects of IL-18 inhibition by this antibody in central nervous system (CNS) injury remain unclear. Therefore, the present study aimed to determine whether IL-18 inhibition via this antibody could exert neuroprotective effects following SCI in mice. Here, we demonstrate that inhibiting IL-18 significantly promotes motor function recovery following SCI by reducing glial activation, neuronal death, and inflammatory cytokines while promoting the anti-inflammatory M2 polarization of microglia/macrophages.

## 2. Materials and Methods

All experiments were conducted in more than triplicate to ensure reproducibility.

### 2.1. Mice

Eight-week-old C57BL/6J mice were obtained from Japan SLC, Inc. (Shizuoka, Japan) and were bred and maintained at the Institute of Experimental Animal Sciences, Shimane University Graduate School of Medicine. All efforts were made to minimize animal use during the experimental procedures. This study was approved by the Institutional Animal Care and Use Committee of Shimane University, and all experiments were conducted in accordance with the Guide for the Care and Use of Laboratory Animals established by Shimane University.

### 2.2. Surgical Procedures for SCI and Anti-IL-18 Antibody Treatment

Mice were anesthetized with a mixture of butorphanol (Vetorphale^®^, 0.5 mg/mL, Meiji Seika Pharma, Tokyo, Japan), midazolam (Dormicum^®^, 0.4 mg/mL, Roche, Mannheim, Germany), and medetomidine (Domitor^®^, 0.03 mg/mL, Orion Pharma) via intraperitoneal injection. SCI was performed as previously described [24]. Briefly, the connective and muscle tissues were removed to expose the lower thoracic spinal cord. Following T8 laminectomy, a dorsal hemisection was performed at T8 using a surgical blade to a depth of 1.0 mm. To avoid damaging the remaining portion of the lateral CST, the surgical blade was passed through the dorsal spinal cord several times, creating a lesion that extended downward to the central canal. All mice exhibited complete paralysis of both hindlimbs following the surgery. The anti-IL-18 blocking monoclonal antibody 5-4.1, which specifically recognizes a neoepitope of caspase-1/4-cleaved IL-18, was generated as previously described [17]. The IL-18 antibody was administered via intraperitoneal injection according to the schedule.

### 2.3. Behavioral Tests

Behavioral tests were performed as previously described [24,25]. All animals were subjected to five motor tests to evaluate functional recovery after SCI: the Basso Mouse Scale (BMS), ladder walk, and rotarod tests. To evaluate normal performance, baseline scores for each animal were recorded just before surgery.

#### 2.3.1. BMS Score

Previous studies have indicated that the BMS score correlates with hindlimb motor function [26]. BMS scores were determined at the following time points: 1, 3, 7, 14, 21, 28, 35, and 42 days post-injury. The average scores of the right and left hindlimbs were used.

#### 2.3.2. Ladder Walk Test

The ladder walk test was used to assess precise limb placement and stepping while walking along a horizontal ladder with variable rung spacing [27]. The ladder was designed as previously described [28]. The mice received training three times per session the day before the injury, and the percentage of foot slips for each hind paw was recorded. Testing began two weeks after injury and was performed once a week for an additional four weeks. The average scores of the right and left hindlimbs were used.

#### 2.3.3. Rotarod Test

The rotarod test was used to assess motor recovery in rodents after SCI [29,30]. The animals were placed on a rotating rod (30 mm diameter) that gradually accelerated from 0 to 50 r.p.m. over 5 min. Mice were trained three times a day for three days before injury. The total time on the rod was recorded until the mouse either fell off or gripped and spun around the rod two times. The baseline value (pre-injury) was scored as the mean of three trials, recorded one day before SCI.

### 2.4. Immunohistochemistry

Spinal cord sections were prepared three days after SCI. The mice were transcardially perfused with PBS, followed by 4% paraformaldehyde in 0.1 M phosphate buffer. The spinal cords were dissected, post-fixed in the same fixative, and immersed overnight in PBS containing 30% sucrose. Tissues were then embedded in the Tissue-Tek OCT compound and frozen at −80 °C until use. Sections (20 µm thick) were cut on a cryostat and mounted on Matsunami adhesive (MAS)-coated slides (Matsunami, Osaka, Japan) [24]. Cryostat sections were incubated with a blocking solution containing 5% BSA and 0.1–0.3% Triton X-100 in PBS for 1 h at room temperature, followed by overnight incubation with the indicated primary antibodies at 4 °C: anti-Iba1 (Wako, Osaka, Japan), anti-GFAP antibody (Sigma, St. Louis, MO, USA), and anti-NeuN antibody. Alexa Fluor 488- or 568-conjugated secondary antibodies (Molecular Probes, Eugene, OR, USA) were applied for 1 h at room temperature. Immunoreactivity was visualized using fluorescence microscopy. Samples were coverslipped with a mounting medium (DAKO Corporation, Carpinteria, CA, USA) and examined under a fluorescence microscope (Olympus BX53, DP71).

### 2.5. Cytokine Array

For the cytokine array, the mouse cytokine array panel A (R&D Systems, Minneapolis, MN, USA) was used. Spinal cord lesion sites were collected three days after SCI, as indicated. Tissue lysates were prepared in 1% Triton X-100 in PBS containing a protease inhibitor cocktail (Roche Diagnostics, Mannheim, Germany) at 4 °C. The lysates were frozen, thawed, and centrifuged at 10,000× *g* for 5 min to remove cellular debris. An aliquot of the sample was taken, and protein concentrations were quantified using a total protein assay. The lysates were stored at −80 °C and thawed on ice before use. The tissue lysates were incubated with the cytokine array membrane according to the manufacturer’s protocol. Signals were analyzed using the Amersham Imager system (GE Healthcare, Madison, WI, USA), and the signal intensities were quantified using the accompanying software. The results were compared to those from the control antibody-treated group.

### 2.6. RNA Extraction, Reverse Transcription, and Real-Time PCR

Total RNA was extracted from motor cortex samples using TRIzol (Invitrogen, Carlsbad, CA, USA) and reverse-transcribed using ReverTra Ace qPCR RT Master Mix with gDNA Remover (TOYOBO, Osaka, Japan). A real-time PCR determined the mRNA expression (CronoSTAR96 Real-Time PCR System; Clontech, Palo Alto, CA, USA). For SYBR Green assays, 10 μL reactions contained 1× iTaq Universal SYBR Green Supermix (Bio-Rad, Hercules, CA, USA), 400 nM gene-specific primers, and a 1 μL template. The PCR cycles began with UNG digestion at 50 °C for 2 min, initial denaturation at 95 °C for 10 min, followed by 45 cycles of 95 °C for 15 s and annealing at 60 °C for 1 min. A gradual temperature increase from 60 °C to 95 °C during the dissociation stage confirmed primer specificity.

Relative mRNA expression was normalized to the internal control. Cycle threshold (Ct) values were calculated using the ΔΔCt method to determine fold differences.

### 2.7. Statistical Analysis

Statistical analyses are described in the figure legends. Data are presented as mean ± S.E. from at least three independent experiments, and *p*-values less than 0.05 were considered significant.

## 3. Results

### 3.1. Increased IL-18 Expression After SCI

**Figure 1:**
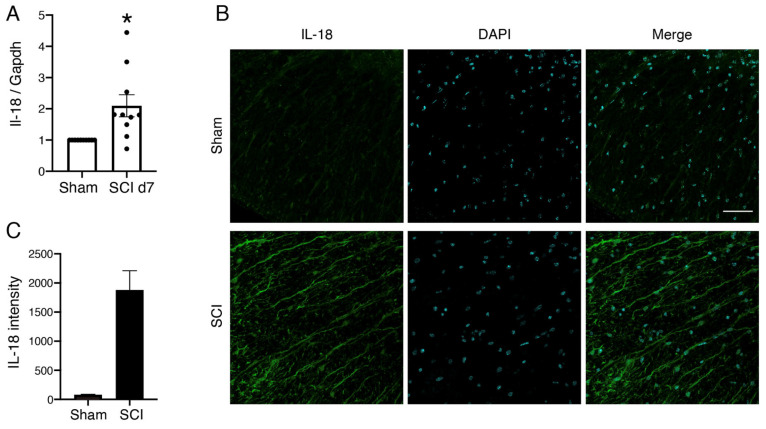
Changes in IL-18 expression following SCI. (**A**) IL-18 expression levels in the spinal cord 7 days after SCI. Relative IL-18 mRNA levels were quantified using real-time PCR and normalized to GAPDH. Data are presented as the mean ± SE from three independent experiments (*n* = 10). Results are shown as fold changes compared to sham mice. * *p* < 0.05; analysis of variance (ANOVA) with Welch’s *t*-test. (**B**) Distribution of IL-18 (green) in the spinal cord 3 days after SCI. (**C**) IL-18 signal intensity as shown in (**B**). DAPI (cyan) was used for nuclear staining. Scale bar, 50 μm. SCI: spinal cord injury; GAPDH: glyceraldehyde 3-phosphate.

We first examined whether IL-18 expression was affected by SCI. In our SCI model, the mice underwent a dorsal hemisection at T7. A 1 cm segment of spinal cord tissue centered on the lesion epicenter was analyzed. IL-18 RNA expression significantly increased in the spinal cord 7 days after SCI (Figure 1A), suggesting that IL-18 is involved in the physiological processes following SCI.

We also examined the distribution of IL-18 in the spinal cord following injury. IL-18 expression was observed in the white matter of the spinal cord 3 days after SCI (Figure 1B).

### 3.2. IL-18 Inhibition-Enhanced Behavioral Recovery Following SCI

**Figure 2:**
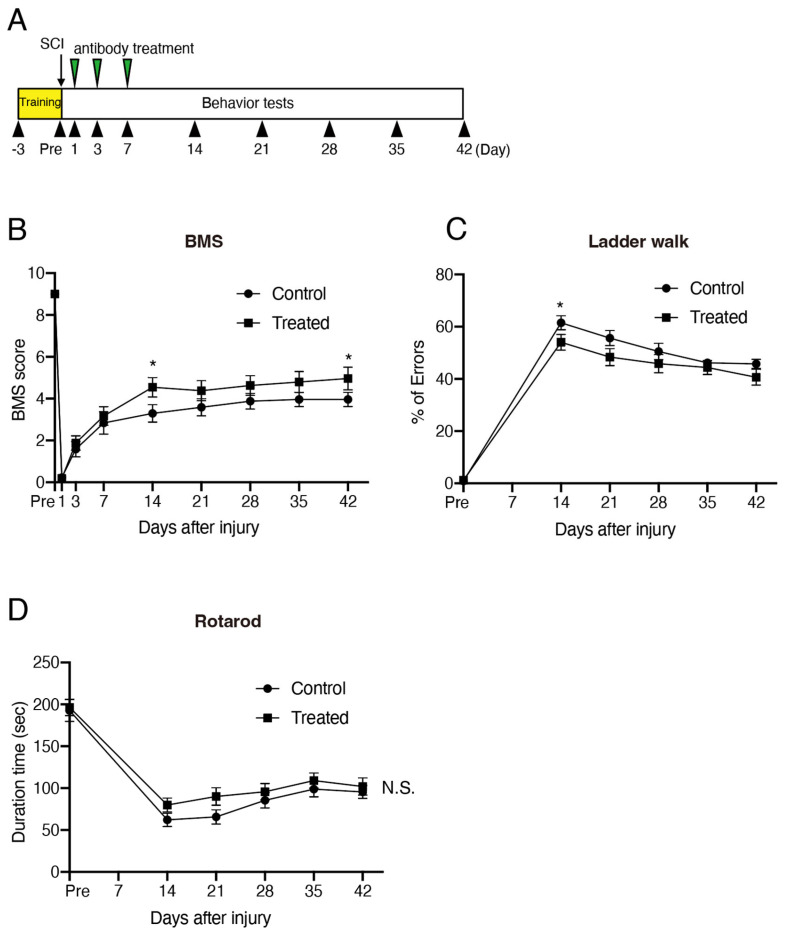
Inhibition of IL-18 improves recovery of motor function following SCI. (**A**) Experimental schedule. (**B**) BMS scores were significantly higher in IL-18 antibody-treated mice compared to control antibody-treated mice. (**C**) IL-18 antibody treatment reduced errors in the ladder walk test. (**D**) No significant difference was observed in the rotarod test. Results are presented as mean ± SE (*n* = 12 per group). * *p* < 0.05; two-way repeated-measures ANOVA with Sidak’s multiple comparisons test. SCI: spinal cord injury; BMS: Basso Mouse Scale; N.S.: not significant.

We next assessed the functional effect of IL-18 blockade after SCI. Since thoracic spinal cord injury primarily impairs motor function below the lesion site, we evaluated hindlimb motor function. The Basso Mouse Scale for Locomotion (BMS), widely used to assess locomotor recovery in mouse SCI models [26], was employed. The mice were intraperitoneally treated with a control or IL-18 antibody at 1, 3, and 7 days after SCI (Figure 2A). Both control- and IL-18 antibody-treated mice exhibited impaired BMS scores one day after SCI (Figure 2A). By 14 days after SCI, IL-18 antibody-treated mice displayed higher BMS scores than controls (Figure 2B). To verify this result, we further assessed hindlimb functional recovery using additional motor tests. Footfall frequency during walking was evaluated with the ladder walking test, where the IL-18 antibody-treated mice exhibited significantly improved functional recovery compared to the controls at 14 days after SCI (Figure 2C). However, IL-18 antibody-treated mice did not show significantly better performance in the rotarod test following injury (Figure 2D). These results suggest that IL-18 inhibition contributes to functional recovery, particularly for tasks requiring precise limb coordination, such as the ladder walking test, during the early stages after SCI.

### 3.3. IL-18 Inhibition Attenuates Neuronal Loss After SCI

**Figure 3:**
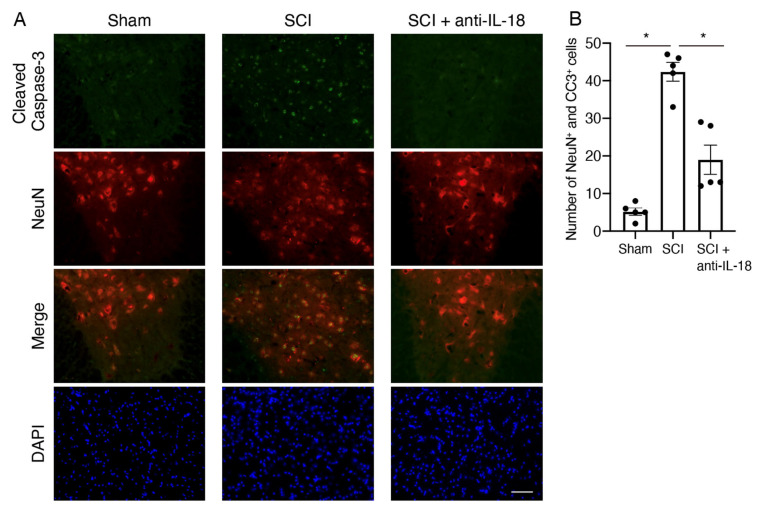
Inhibition of IL-18 ameliorates the death of NeuN-positive neurons following SCI. (**A**) Representative images of immunohistochemical staining of neuronal cell death in the gray matter following SCI. Neurons were immunostained with an anti-NeuN antibody (red), and apoptotic cells were detected using an anti-cleaved caspase-3 (CC3) antibody (green). Scale bar: 50 μm. (**B**) Quantification of NeuN- and CC3-positive cells in the gray matter 2 mm rostral to the lesion epicenter, 7 days after SCI. Results are mean ± SE of five mice per group. * *p* < 0.05; ANOVA with Tukey’s multiple comparisons test. Scale bar: 50 μm. SCI: spinal cord injury; NeuN: neuronal nuclei; CC3: cleaved caspase-3.

Since our findings support that IL-18 inhibition promotes motor functional recovery, we further investigated whether IL-18 antibody suppresses neuronal loss after SCI. As motor function recovery was observed from 14 days post-SCI (Figure 2B,C), we focused on the effect of IL-18 antibodies on neurons before this phase. To determine its effect on motor neuron survival, co-staining with NeuN (red fluorescence) and cleaved caspase-3 (green fluorescence) was performed 7 days post-SCI (Figure 3A). The number of cleaved caspase-3-positive cells in the ventral horn significantly increased in the SCI group, but IL-18 antibody treatment attenuated this effect (Figure 3A). The number of cleaved caspase-3-positive and NeuN-positive neurons in the ventral horn also increased following SCI, whereas IL-18 antibody treatment markedly reduced this effect (Figure 3B). These results suggest that IL-18 inhibition reduces neuronal cell death after SCI.

### 3.4. IL-18 Inhibition Attenuates Gliosis After SCI

**Figure 4:**
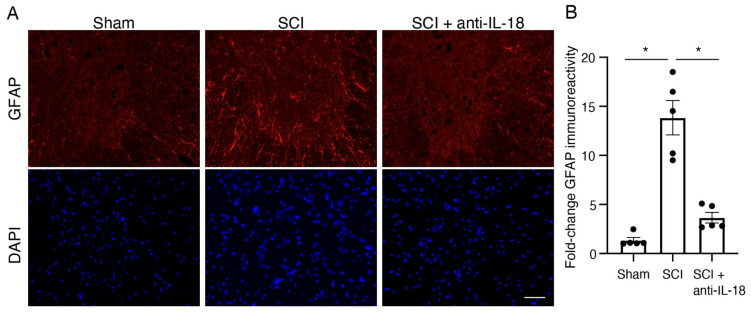
Treatment of IL-18 antibody alleviates reactive gliosis. (**A**) Representative images of GFAP-positive cells (red) labeled with immunofluorescence 3 days after SCI. (**B**) Quantification of GFAP immunoreactivity in a 200× magnification field. Results are mean ± SE of five mice per group. * *p* < 0.05; ANOVA with Tukey’s multiple comparisons test. Scale bar: 50 μm. SCI: spinal cord injury; GFAP: glial fibrillary acidic protein.

To analyze reactive astrogliosis-mediating glial scar formation [31], GFAP immunoreactivity was assessed 3 days after SCI. The GFAP immunoreactivity was significantly elevated after SCI (Figure 4A). GFAP-positive astrocytes following SCI were hypertrophied, with thickened branches, demonstrating morphological changes associated with reactive astrocytes. Compared to the control antibody-treated group, the average fluorescence intensity of GFAP in the IL-18 antibody-treated group was reduced (Figure 4B). These findings indicate that IL-18 inhibition attenuates astrocyte activation and gliosis.

### 3.5. IL-18 Inhibition Attenuates the Accumulation of Microglia/Macrophages After SCI

**Figure 5:**
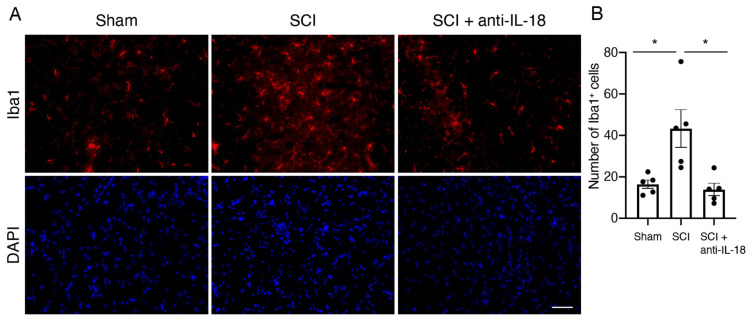
Inhibition of IL-18 attenuates microglia/macrophage activation. (**A**) Representative images of Iba1-positive cells (red) labeled with immunofluorescence 3 days after SCI. (**B**) Quantification of Iba1-positive cells in a 200× magnification field. Results are mean ± SE of five mice per group. * *p* < 0.05; ANOVA with Tukey’s multiple comparisons test. Scale bar: 50 μm. SCI: spinal cord injury; Iba1: ionized calcium-binding adapter molecule 1.

Microglia/macrophages are the main effectors of neuroinflammation after injury [32]. To analyze these cells, we performed immunostaining with an anti-Iba1 antibody (Figure 5A). The number of Iba1-positive microglia/macrophages in the SCI group was significantly higher than in the sham group 3 days after SCI (Figure 5B). This increase in Iba1-positive cells was remarkably reversed by IL-18 antibody treatment.

**Figure 6:**
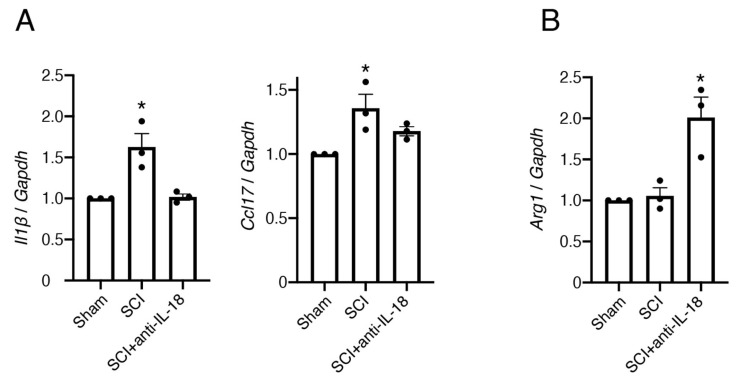
Inhibition of IL-18 attenuates the pro-inflammatory (M1) response and increases the anti-inflammatory (M2) response. (**A**) IL-18 antibody reduces pro-inflammatory cytokine levels and the M1 microglia/macrophage marker. Relative mRNA expression levels of IL-1β and Ccl17 in the spinal cord 3 days after SCI were measured by qPCR. (**B**) IL-18 antibody increases anti-inflammatory cytokine levels and the M2 microglia/macrophage marker. Relative mRNA expression levels of Arg1 in the spinal cord 3 days after SCI were measured by qPCR. All data are presented as mean ± SE, *n* = 4. * *p* < 0.05; ANOVA with Dunnett’s multiple comparisons tests. SCI: spinal cord injury; Arg1: arginase1; GAPDH: glyceraldehyde 3-phosphate.

Based on previous studies, we performed a qPCR to test the transcriptional levels of the markers for the M1 phenotype microglia/macrophages. The expression of the pro-inflammatory cytokine IL-1β and the M1-type microglia/macrophage marker Ccl17 increased following injury, but IL-18 antibody treatment reduced the expression of these genes (Figure 6A). Furthermore, IL-18 antibody treatment significantly increased the expression of the M2-type microglia/macrophage marker arginase 1 (Arg1) (Figure 6B). These results suggest that IL-18 inhibition promotes the polarization of M1 microglia/macrophages to the M2 phenotype and attenuates inflammatory responses.

### 3.6. IL-18 Inhibition Reduces Pro-Inflammatory Cytokine Levels

**Figure 7:**
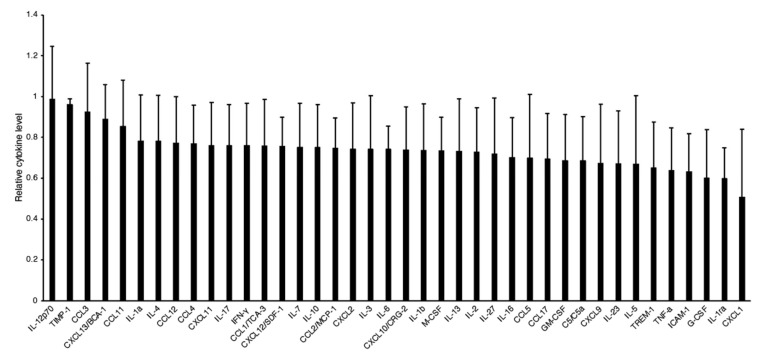
Cytokine/chemokine expression in the spinal cord of IL-18 antibody-treated mice. Pro-inflammatory cytokine levels, including TNF-α and CXCL1, showed a decreasing trend in IL-18 antibody-treated mice compared to control-treated mice.

To further investigate whether IL-18 antibody suppresses the inflammatory response after SCI, we examined cytokine levels at the lesion site of the spinal cord using a cytokine/chemokine array. Inflammatory cytokines, such as tumor necrosis factor-α (TNF-α), tended to decrease in the IL-18 antibody-treated mice compared to the control antibody-treated mice (Figure 7). The C-X-C motif chemokine ligand 1 (CXCL1), which regulates monocyte/macrophage infiltration, also showed a decreasing trend. Additionally, there was a tendency for a decreased expression of the triggering receptor expressed on myeloid cells (TREM-1), an immunoglobulin receptor in neutrophils and monocytes involved in amplifying acute inflammatory responses, although these differences were not statistically significant.

## 4. Discussion

This study demonstrated that inhibiting IL-18 with a blocking antibody significantly improved motor functional recovery following SCI by reducing neuroinflammation, glial activation, and neuronal cell death. We also found that the IL-18 antibody reduced glial activation and neuronal cell death around the injury site. These findings suggest that this antibody represents a promising approach for alleviating neurological symptoms post-injury, which is consistent with previous studies on IL-18 binding protein (IL-18BP), a natural antagonist of IL-18 [15,16].

Our observations revealed an increase in IL-18 expression in the spinal cord at 7 days post-injury [33,34,35,36]. This increased expression was predominantly concentrated in the white matter, with a scattered distribution in the gray matter. Previous reports indicate that both IL-18 and its receptor subunits (IL-18R) are inducible, and their levels in the CNS increase under pathological conditions. For instance, IL-18 and IL-18R are upregulated in the hyperactive microglia and astrocytes, respectively [37,38]. Similarly, hypoxic–ischemic brain injury markedly increased IL-18 expression in the mouse microglia [39]. Treatment with IL-18BP, a natural IL-18 antagonist, significantly improved neurological recovery after traumatic brain injury [40]. By binding to active IL-18, IL-18BP prevents its interaction with IL-18 receptors, effectively neutralizing its pro-inflammatory signaling cascade. This regulatory mechanism not only mitigates inflammation but also positions IL-18BP as a potential biomarker for assessing inflammation severity in neuroinflammatory conditions. Clinical and preclinical studies have highlighted IL-18BP’s therapeutic relevance in conditions such as traumatic brain injury, multiple sclerosis, and systemic inflammatory disorders. Its natural antagonistic role reinforces the rationale for targeting IL-18 signaling in spinal cord injuries and underscores the broader applicability of IL-18 blockade in the treatment of neuroinflammation and neurodegeneration. These observations prompted us to investigate the effects of IL-18 inhibition on glial cells. Reactive astrocytes contribute to glial scar formation following SCI, creating a physical and biochemical barrier to axon regeneration and impacting neurological functional recovery [41]. Attenuation of reactive astrocytes has been shown to promote functional recovery after SCI. Here, we demonstrated that treatment with the IL-18 blocking antibody significantly reduced GFAP immunoreactivity and decreased the number of Iba1-positive cells, suggesting that IL-18 inhibition suppresses the activation of astrocytes and microglia/macrophages. Our findings are consistent with previous studies implicating IL-18 dysregulation in neuroinflammatory processes and neurological disorders. However, it is important to note that the precise mechanisms underlying the IL-18-mediated effects in SCI may vary depending on the specific context and experimental model. The present study extends the understanding of IL-18’s role in SCI pathophysiology by elucidating its contributions to glial activation, neuronal death, and functional recovery post-injury.

The microglia have been shown to express IL-18R, with IL-18 possibly acting on the microglia through an autocrine mechanism to sustain their activation [42]. Following MPTP treatment, IL-18 contributes to the maintenance of microglial activation. Additionally, IL-18 is expressed in the microglia and promotes their sustained activation after peripheral nerve injury, potentially leading to neuropathic pain [37]. Following peripheral nerve injury, IL-18 expression significantly increases in spinal microglia and persists for an extended period. Intrathecal injection of IL-18 into naïve rats induces tactile allodynia, a hallmark of neuropathic pain, and activates both the microglia and astrocytes. Inhibiting IL-18 signaling with antibodies or IL-18BP, a natural inhibitor, reduces tactile allodynia. These findings suggest that IL-18 contributes to microglial activation across various experimental models, indicating common underlying mechanisms.

IL-18BP binds to active IL-18, neutralizing its pro-inflammatory effects. In models with elevated endogenous IL-18BP levels, the effects of IL-18 antibodies may be partly modulated by this natural buffering mechanism. Notably, IL-18BP levels increase in various inflammatory and neurodegenerative conditions, which can influence the efficacy of exogenous IL-18 inhibitors. Future studies should quantify IL-18BP levels in the spinal cord following injury and assess how its interaction with IL-18 antibodies impacts functional outcomes.

Increased inflammatory responses lead to neurotoxic damage. Our results show that inhibiting IL-18 decreases neuronal death (Figure 3) and the tendencies of the decreased cytokine/chemokine expression, such as interferon-γ (IFN-γ) and tumor necrosis factor-α (TNF-α) (Figure 7). Consistently, the inhibition of the NLRP3 inflammasome, which activates caspase-1 to promote the maturation and release of IL-18, has also been shown to reduce inflammatory cytokines after traumatic brain injury [43]. The reduced neuronal death in IL-18 antibody-treated mice suggests that IL-18 contributes to neuronal damage, likely through microglial activation. Our qPCR results demonstrated increased expression of IL-1β and Ccl17 following SCI. The inhibition of IL-18 reduces IL-1β and Ccl17 expression and is accompanied by an increase in Arg1 expression. These findings suggest that the IL-18 blocking antibody promotes the transformation of M1 microglia/macrophages to the M2 phenotype.

Moreover, the results from Figure 6 provide a direct mechanistic link between IL-18 inhibition and M2 polarization. By decreasing the M1 markers (e.g., IL-1β, Ccl17) and significantly increasing the M2 marker Arg1, IL-18 inhibition appears to foster an anti-inflammatory and neuroprotective microenvironment. This polarization likely contributes to improved motor functional recovery following SCI. These observations align with prior studies highlighting the therapeutic potential of targeting IL-18 in CNS injury contexts. Additionally, while behavioral and molecular assessments were robust, the absence of longitudinal imaging or electrophysiological data limits a comprehensive understanding of the long-term neuroprotective effects of IL-18 inhibition.

IL-18 antibody treatment alleviates, but does not fully reverse, the motor deficits induced by SCI. The observed differences in outcomes among the ladder walk test, the BMS test, and the Rotarod test in your SCI model can be attributed to the distinct motor functions and neurological pathways assessed by each test. The ladder walk test and the BMS test primarily evaluate gross motor coordination, limb placement, and weight-bearing capabilities. Specifically, the ladder walk test emphasizes skilled walking and stepping patterns, which require precise limb placement and adaptive responses to irregular rung spacing. This test is particularly sensitive to subtle impairments in motor coordination and compensatory movements, making it highly relevant for assessing recovery in SCI studies. Similarly, the BMS test assesses open-field locomotion, evaluating hindlimb movements, coordination, and body support, thereby providing a broad measure of functional recovery post-injury. The differences were observed at 14 days post-injury, and it seems a period of 14 days is critical for the recovery detected by the BMS test and the ladder walk test. The Rotarod test, in contrast, measures motor endurance, balance, and grip strength, which are more dependent on cerebellar function and overall neuromuscular endurance. While motor deficits may recover sufficiently to improve basic locomotion, as evidenced by performance in the ladder walk and BMS tests, they may not fully restore the motor balance or sustained grip strength required for Rotarod performance. This difference suggests that, although anti-IL-18 antibody treatment facilitates recovery in tasks involving basic locomotion, such as those evaluated in the ladder walk and BMS tests, it may not comprehensively address the neurological or muscular deficits underlying balance and endurance challenges in the Rotarod test. Further investigation into these limitations, including the effects of the IL-18 antibody on specific motor pathways and potential optimization strategies, is needed to elucidate the precise mechanisms.

One limitation of this study is the reliance on a single dose and administration route for the IL-18 antibody treatment. The optimal dosage of the IL-18 antibody likely varies across different disease animal models. Future studies could explore dose–response relationships using varied concentrations of the IL-18 antibody to identify the optimal therapeutic window for SCI recovery. In prior studies, we demonstrated the antibody’s efficacy in mouse models of colitis when administered intraperitoneally [22,23]. Based on these findings, we selected an intraperitoneal dose of 150 μg for this study. While this dose improved motor functional recovery in the SCI model, it remains unclear if it represents the optimal dosage for SCI. Additionally, alternative administration routes, such as localized delivery, could potentially yield varying therapeutic outcomes. Optimizing dosage and administration routes specifically for the SCI model may enhance the therapeutic efficacy and applicability of long-term treatments.

Future research should aim to optimize the dosing and delivery of the IL-18 antibody to achieve maximal efficacy. Investigations into the long-term effects of IL-18 inhibition, including its potential impacts on neuroregeneration and chronic inflammation, are also warranted. The comparison of IL-18 expressions across different time spans would be helpful to address this point. Furthermore, combining IL-18 inhibition with other therapeutic strategies, such as anti-fibrotic agents or neuro-regenerative treatments, could offer a synergistic approach to mitigating the complex secondary injury cascade following SCI.

## 5. Conclusions

In conclusion, antibody-mediated downregulation of IL-18 ameliorates neuroinflammation and improves functional recovery in mice with SCI. We demonstrated that IL-18 inhibition promotes the conversion of microglia/macrophages from a pro-inflammatory to an anti-inflammatory phenotype and prevents neuronal loss following SCI. Additionally, the IL-18 blocking antibody reduced astrocyte activation, further preventing neuronal death. While our findings highlight a clear association between IL-18 inhibition and improved outcomes following SCI, several limitations warrant consideration. For example, further studies are needed to elucidate the underlying mechanisms driving these effects. Moreover, the current study primarily focused on short-term outcomes, leaving the long-term effects of IL-18 blockade to be clarified. Investigations into the optimal dosing and treatment windows for IL-18 inhibition could enhance therapeutic efficacy. Future studies addressing these points will further underscore the potential utility of IL-18 targeted therapies in the improvement of functional recovery and the mitigation of secondary injury cascades following neurological insults.

## Data Availability

The original contributions presented in this study are included in the article. Further inquiries can be directed to the corresponding author.
